# A novel cystathionine γ-lyase inhibitor, I194496, inhibits the growth and metastasis of human TNBC via downregulating multiple signaling pathways

**DOI:** 10.1038/s41598-021-88355-9

**Published:** 2021-04-26

**Authors:** Ya Liu, Lupeng Wang, Xiuli Zhang, Yuying Deng, Limin Pan, Hui Li, Xiaoyan Shi, Tianxiao Wang

**Affiliations:** 1 School of Pharmacy, Henan University, Jinming Road, Kaifeng, 475004 Henan People’s Republic of China; 2 Department of Botany, Liaoning Agricultural College, Yingkou, 115009 Liaoning People’s Republic of China; 3 School of Basic Medical Sciences, Joint National Laboratory of Antibody Drug Engineering, Henan University, Kaifeng, 475004 Henan People’s Republic of China

**Keywords:** Biochemistry, Biological techniques, Biotechnology, Cancer, Cell biology, Drug discovery, Molecular biology, Oncology

## Abstract

Triple-negative breast cancer (TNBC) is a high-risk subtype of breast cancer with high capacity for metastasis and lacking of therapeutic targets. Our previous studies indicated that cystathionine γ-lyase (CSE) may be a new target related to the recurrence or metastasis of TNBC. Downregulation of CSE could inhibit the growth and metastasis of TNBC. The purpose of this study was to investigate the activity of the novel CSE inhibitor I194496 against TNBC in vivo and in vitro. The anticancer activity of I194496 in vitro were detected by MTS, EdU, and transwell assays. Methylene blue assay was used to determine the H_2_S level. Western blot was performed to analyze the expression of related pathway proteins. Xenograft tumors in nude mice were used to analyze the anticancer activity of I194496 in vivo. I194496 exerted potent inhibitory effects than l-propargylglycine (PAG, an existing CSE inhibitor) on human TNBC cells and possessed lower toxicity in normal breast epithelial Hs578Bst cells. I194496 reduced the activity and expression of CSE protein and the release of H_2_S in human TNBC cells. Meanwhile, the protein levels of PI3K, Akt, phospho (p)-Akt, Ras, Raf, p-ERK, p-Anxa2, STAT3, p-STAT3, VEGF, FAK, and Paxillin were decreased in human TNBC cells administrated with I194496. Furthermore, I194496 showed more stronger inhibitory effects on human TNBC xenograft tumors in nude mice. I194496 could inhibit the growth of human TNBC cells via the dual targeting PI3K/Akt and Ras/Raf/ERK pathway and suppress the metastasis of human TNBC cells via down-regulating Anxa2/STAT3 and VEGF/FAK/Paxillin signaling pathways. CSE inhibitor I194496 might become a novel and potential agent in the treatment of TNBC.

## Introduction

Triple negative breast cancer (TNBC) is a subtype of breast cancer with the highest metastatic rate and the lowest overall survival rate^1–3^. Moreover, TNBC is hard to target treatment due to the lack of estrogen receptors (ER), progesterone receptors (PR) and human epidermal growth factor receptor 2 (HER2). At present, the only available strategy for TNBC is still challenging the chemical treatment^4^. However it is limited by the toxicity and multi-drug resistance of chemotherapy drugs^5^. Consequently, an effort to develop targeted therapies for TNBC is crying need.

Hydrogen sulfide (H_2_S) is the third gaseous signaling molecule, along with nitric oxide^6–10^ and carbon monoxide^11,12^, not only functions in numerous physiological and developmental processes, but also serves important roles in cancer^13,14^. Cystathionine γ-lyase (CSE) is an endogenous enzyme that produces H_2_S. Researches have shown that endogenous H_2_S produced by CSE could promote the growth of human tumor cells^15–17^. Our previous studies indicated that the high-expression of CSE protein promoted the proliferation and metastasis of TNBC MDA-MB-231 cells^18,19^. Therefore, CSE may be a new target for the metastasis of TNBC. The research on inhibitors targeting CSE will be of great significance for the treatment of TNBC.

I194496, a new inhibitor targeting CSE protein, was obtained through virtual screening according to the crystal structure of CSE protein by our research group. In this study, we firstly in vitro determined the roles of I194496 in human TNBC. We then detected the effects of I194496 on tumor growth in nude mice bearing human TNBC xenografts. This study will make it possible to find new ways in how to get to target therapy of TNBC and how to overcome the recurrence or metastasis of TNBC.

## Materials and methods

### Compound I194496 and l-propargylglycine (PAG)

**Figure 1:**
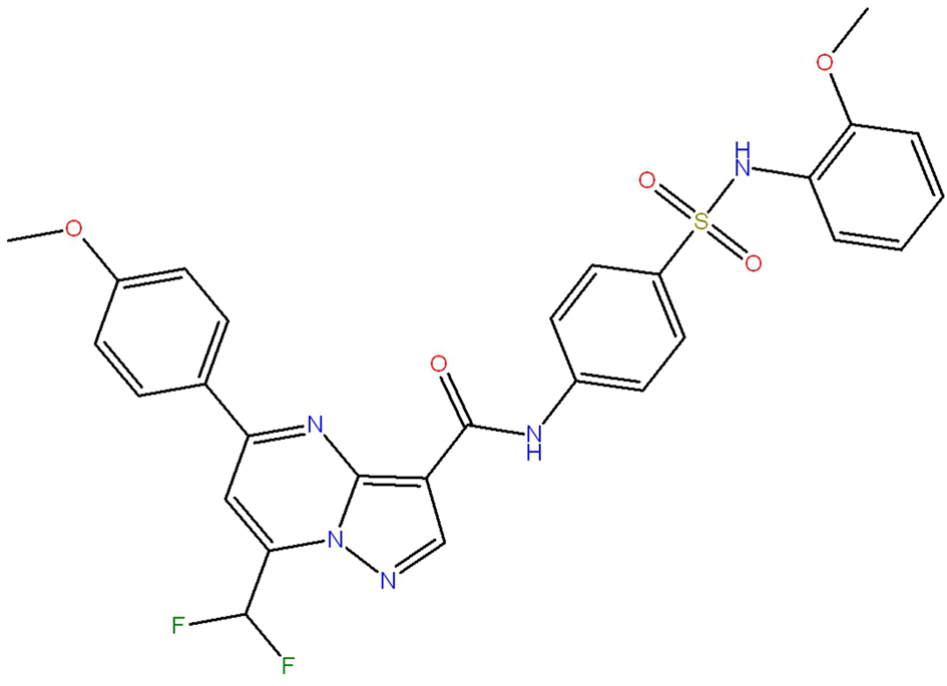
Structure of I194496. I194496 was known as 7-(difluoromethyl)-N-{4-[(2-methoxyanilino) sulfonyl]phenyl}-5-(4-methoxyphenyl)pyrazolo[1,5 a]pyrimidine-3-carboxamide.

I194496, a novel CSE inhibitor, was known as 7-(difluoromethyl)-N-{4-[(2-methoxyanilino) sulfonyl]phenyl}-5-(4-methoxyphenyl)pyrazolo[1,5-a]pyrimidine-3-carboxamide and purchased from Specs (Zoetermeer, The Netherlands). The chemical structure of I194496 is shown in the Fig. 1. The binding mode of I194496 and CSE protein was shown in our pervious study^20^. l-Propargylglycine (PAG), an existing CSE inhibitor, was purchased from Sigma-Aldrich; Merck KGaA (Darmstadt, Germany). PAG was dissolved in normal saline. I194496 was dissolved in DMSO as 50 mM and then diluted in cell culture to the desired concentration, with a,final DMSO concentration of less than 0.1%.

### Cell lines

Human TNBC cell lines MDA-MB-231 and MDA-MB-468, and human normal breast epithelial cell line Hs578Bst were purchased from the ATCC (Manassas, VA, USA). The cells were cultured in Dulbecco's modified Eagle's medium (DMEM; Thermo Fisher Scientific, Inc., Waltham, MA, USA) supplemented with 10% fetal bovine serum (FBS; Zeta Life, Inc., San Francisco, CA, USA) at 37 °C 5% CO_2_.

### qRT-PCR assay

Total RNA was extracted from the cells with Trizol reagent and reverse transcribed with Vazyme RT kit (Nanjing, China) in accordance with a standard protocol.qRT-PCR was performed on an ABI StepOnePlus Real-Time PCR System (Vazyme,Nanjing, China) using ChamQ Universal SYBR Master Mix. The 2^−△△Ct^ method was used to evaluate the mRNA expression levels of CSE gene. The following primers were used in the RT-qPCR assay: CSE (5′-CCCATCTCACTGTCCACCAC-3′; 5′-GTGCTGCCACTGCTTTTTCA-3′), and GAPDH (5′-GCACCGTCAAGGCTGAGAAC-3′; 5′-TGGTGAAGACGCCAGTGGA′).

### Analysis of CSE recombinant protein activity

Each test consisted of a 100 µL reaction mixture containing 5 µg of the purified CSE enzyme, 0.01 mM PLP, 1 mM l-cys and 50 mM sodium phosphate buffer pH 8.2. The inhibitors were added to the reaction 15 min before l-cys was added to the solution. Reaction was initiated by transferring the Eppendorf tubes from ice to a 37 °C shaking water bath. After 60 min of incubation at 37 °C, the reaction was terminated by adding 1% ZnAc to trap H2S followed by 10% TCA to precipitate proteins. Subsequently, *N,N*-dimethyl-*p*-phenylenediamine-sulfate was immediately followed by addition of Ammonium ferric sulfate.The absorbance of the resulting solution was measured at 670 nm. The percentage of H_2_S was calculated as 100% in the group without inhibitor.

### Detection of endogenous H_2_S level in TNBC cells

H_2_S production was determined with the methylene blue method and the procedures referred to the reference^20^. The level of H_2_S was calculated according to the absorbance at 670 nm and was presented as nmol min^−1^ per 1 × 10^6^ cells. The assay was repeated in three independent experiments.

### MTS assay

MTS assay was used to evaluate the effects of different concentrations of I194496 on cell viability of TNBC cells. MDA-MB-231 and MDA-MB-468 cells were plated into 96-well plates at a density of 1 × 10^5^/ml (100 µL/well), and were separately treated with 0, 20, 30 and 40 µM of I194496 and 0, 24, 28 and 32 µM of I194496 for 48 h at 37 °C in 5% CO_2_. And then MTS was added and continued for 4 h at 37 °C in 5% CO_2_, followed by reading the absorbance at 490 nm. 40 µM of PAG served as control. The assay was repeated in three independent experiments.

### 5-Ethynyl-2′-deoxyuridine (EdU) assay

EdU assay was performed to assess the effects of I194496 on proliferation of TNBC cells. MDA-MB-231 and MDA-MB-468 cells (1 × 10^5^/ml, 100 µl/well) were plated in 96-well plates and cultured for 24 h, and then were respectively exposed to 0, 20, 30 and 40 µM I194496 and 0, 24, 28 and 32 µM of I194496 for 24 h at 37 °C. Cell proliferation was investigated with the EdU assay kit (Guangzhou Ribobio Co., Ltd., Guangzhou, China). The procedures were performed according to the references^19,20^.

### Transwell assay

Transwell assay was performed to test the cell migration and invasion. 24-well Transwell chambers (pore size, 8 µm; Corning Incorporated, Corning, NY, USA) was used to perform the assay. Here, the transwell chambers need to be covered with Matrigel matrix (BD Biosciences, CA, USA) for invasion, but not for migration. Briefly, 2.5 × 10^4^ (for migration) or 5 × 10^4^ (for invasion) cells suspended in 200 μl DMEM (with 1% FBS) were seeded into the top chambers, whereas DMEM with 15% FBS was placed into the bottom chambers. After 24 h, the cells in the top chambers were respectively treated with 0, 20, 30 and 40 µM I194496 or 0, 24, 28 and 32 µM of I194496 at 37 °C. After 24 h, the cells on the upper surface of the membrane were removed. The migratory and invasive cells attaching to the lower surface of membrane were fixed, stained and imaged according to the methods of references^18–21^.

### ELISA assay of CSE protein

MDA-MB-231 and MDA-MB-468 cells respectively treated with 20, 30, 40 µM and 24, 28 and 32 µM of I194496 for 48 h were broken up by multigelation followed by centrifuging at 1006.2*g* for 10 min to remove particles and polymers. And then the supernatant was added to enzyme plate for ELISA detection according to the protocol of the human CSE ELISA kit (Camilo Biological. Inc., Nanjing, China). The CSE concentration of each sample was calculated according to the standard curve prepared by using the standard product in the kit.

### Western blot analysis

MDA-MB-231 cells and MDA-MB-468 cells were treated with 20, 30 and 40 µM of I194496 or 24, 28 and 32 µM of I194496 for 24 h. The cells were collected and then lysed on the ice by RIPA buffer (50 mM Tris–HCl, pH 8.0; 150 mM sodium chloride; 1.0% NP-40; 0.5% sodium deoxycholate; and 0.1% SDS) supplemented with 10 μg/ml phenylmethylsulfonyl fluoride (Sigma-Aldrich; Merck KGaA) for 30 min, followed by centrifuging at 12,000 × *g* for 10 min to extract the proteins. The proteins concentrations were determined using the bicinchoninic acid protein quantitative kit (Solarbio Science & Technology Co., Ltd.). The protein samples (40 µg) were separated by SDS-PAGE and transferred onto polyvinylidene difluoride (PVDF) membranes (EMD Millipore), followed by incubating the primary antibodies at 4 °C overnight and secondary antibodies for 2 h at room temperature. Here, the membranes prior to incubating the primary antibodies were cropped to facilitate the hybridisation of antibodies against different antigens. And then the proteins were visualized using an EasyBlot Enhanced Chemiluminescence kit (Sangon Biotech Co., Ltd.) and detected using a FluorChem Q Multifluor system (ProteinSimple). GAPDH was used as a loading control. The primary antibodies were as follows: Akt rabbit monoclonal antibody (1:1000; cat no. 4685), pAkt rabbit monoclonal antibody (1:1000; cat. no. 4060), Ras rabbit monoclonal antibody (1:1000; cat. no. 3965), p44/42 MAPK (Erk1/2) rabbit monoclonal antibody (1:1000; cat. no. 4695), pErk1/2 rabbit monoclonal antibody (1:1000; cat. no. 4376), STAT3 mouse monoclonal antibody (1:1000, cat. no. 9139), p-STAT3 (Tyr705) rabbit polyclonal antibody (1:1000, cat. no. 9131) from Cell Signaling Technology, Inc.; CSE mouse monoclonal antibody (1:100; cat. no. sc-365382) from Santa Cruz Biotechnology, Inc.; VEGF rabbit polyclonal antibody (1:1000; cat. no. 19003-1-AP), ANXA2 rabbit polyclonal antibody (1:1000; cat. no. 11256-1-AP), PI3K p110(beta) rabbit polyclonal antibody (1:1000; cat. no. 20584-1-AP), paxillin rabbit polyclonal antibody (cat. no. 22172-1-AP), FAK rabbit polyclonal antibody (1:1000; cat. no. 12636-1-AP), and RAF rabbit polyclonal antibody (cat. no. 551140-1-AP) from ProteinTech Group, Inc.; pANXA2 rabbit polyclonal antibody (1:1000; cat.no. AF7096) from Affinity Biosciences, Inc.; and GAPDH mouse monoclonal antibody (1:1000; cat. no. AG019) from Beyotime Institute of Biotechnology. Horseradish peroxidase-conjugated goat anti-mouse (1:10,000; cat. no. SA00001-1) and horseradish peroxidase-conjugated goat anti-rabbit (1:10,000, cat. no. SA00001-2) from ProteinTech Group, Inc. were secondary antibodies. Image-J2x software (Rawak Software, Inc.) was used for quantitative analysis^19^.

### Animal study

Animal experiments were approved by Biomedical research ethics committee of Henan University (HUSOM-2018-320). Thirty BALB/c nude mice (female; 4-week-old) were purchased from Beijing Vital River Laboratory Animal Technology Co., Ltd. (Certificate No. SCXK (Jing) 2016–0006, Beijing, China). MDA-MB-231 and MDA-MB-468 cells (5 × 10^6^ cells in 100 μl serum-free medium) were injected into the right flanks of mice through the second breast pad. At 24 h, the mice were randomly divided into three groups (n = 5 per group). Then 200 μM I194496, 200 μM PAG, and solvent (normal saline contains 10% DMSO and 10% Tween 80), were subcutaneously injected near the implanted tumor once every 2 days for 14 days. In the process of experiment, the weight of mice and the volumes of tumor were measured daily after tumor growth. Then according to previous references^22,23^, the tumor volumes were calculated as volume (V) = L × W^2^/2 and the tumor volume doubling time (TVDT) was calculated as TVDT = (T − T_0_) × log 2/log (V2/V1)). At the end of the experiment, mice were sacrificed and tumors were excised and weighted to determine the inhibition rate (IR) of tumor growth.

### Immunohistochemistry (IHC) and western blot assay of tumor tissues

After the mice were sacrificed, tumor tissues were excised and conducted IHC and western blot assay. IHC was conducted as previously described^19^. Tumor tissues were stained with anti-CSE antibody (1:300; cat. no. sc-365382). CSE-positive cells were photographed using a Nikon ECLIPSE Ci-L microscope and CSE-positive expression level was calculated as areal density = IOD/AREA, where IOD represents positive cumulative optical density values and AREA represents the pixel area of the organization. IOD and AREA were analyzed with the Image-pro plus 6.0 (Media Cybernetics, Inc., Rockville, MD, USA). Meanwhile, western blot was performed to detect the level of CSE and VEGF proteins in the tumor tissues. CSE mouse monoclonal antibody (1:100; cat. no. sc-365382, Santa Cruz Biotechnology, Inc.) and VEGF rabbit polyclonal antibody (1:1000; cat. no. 19003-1-AP, ProteinTech Group, Inc.) acted as the specific primary antibodies.

### Statistical analysis

SPSS 17.0 software (SPSS, Inc., Chicago, IL, USA) was used for statistical analysis. The data are expressed as the means ± standard deviation. The differences between groups were analyzed using one-way analysis of variance followed by Bonferroni post hoc test. p < 0.05 was considered statistically significant.

### Ethics approval

Animal experiments were approved by Biomedical Research Ethics Committee of Henan University (HUSOM-2018-320).

### Consent for publication

All authors consent for publication.

### Statement of animal experiments

All experimental procedures conformed to the guidelines of Care and Use of Laboratory Animals of China for animal experimentation. All animal handling methods were carried out in accordance with relevant guidelines and regulations. And the study was carried out in compliance with the ARRIVE guidelines.

## Results

### I194496 suppresses CSE/H_2_S system of TNBC cells

**Figure 2:**
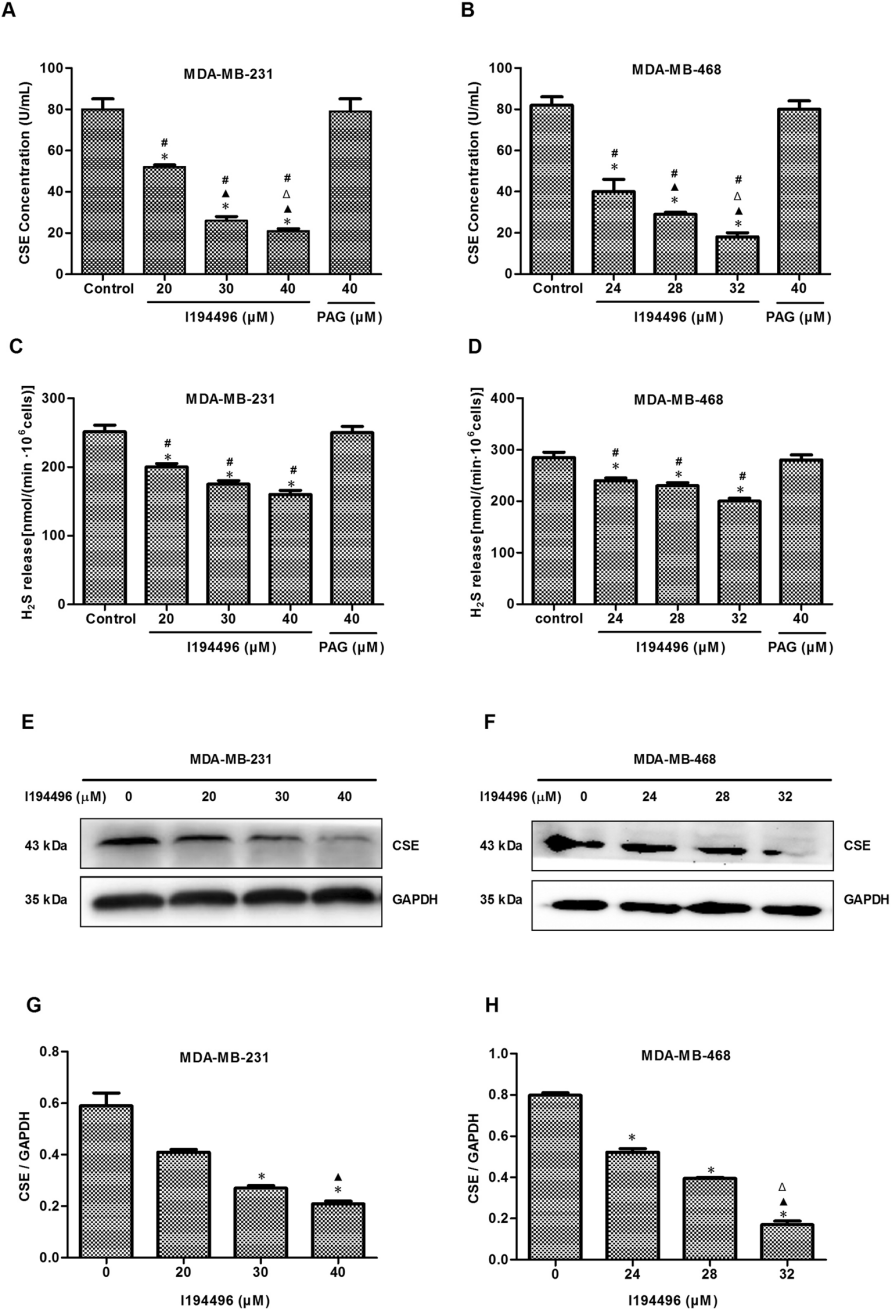
I194496 suppresses CSE/H_2_S system of TNBC cells. (**A**) The effect of I194496 on CSE concentration in MDA-MB-231 cells. *P < 0.05 vs 0 μM I194496 group. ^▲^P < 0.05 vs 20 μM I194496 group. ^Δ^P < 0.05 vs 30 μM I194496 group. ^#^P < 0.05 vs PAG group. (**B**) The effect of I194496 on CSE concentration in MDA-MB-468 cells. *P < 0.05 vs 0 μM I194496 group. ^▲^P < 0.05 vs 24 μM I194496 group. ^Δ^P < 0.05 vs 28 μM I194496 group. ^#^P < 0.05 vs PAG group. (**C**) The effect of I194496 on H_2_S release in MDA-MB-231 cells. *P < 0.05 vs 0 μM I194496 group. ^#^P < 0.05 vs PAG group. (D) The effect of I194496 on H_2_S release in MDA-MB-468 cells. *P < 0.05 vs 0 μM I194496 group. ^#^P < 0.05 vs PAG group. (**E**, **G**) The effect of I194496 on expression of CSE in MDA-MB-231 cells. *P < 0.05 vs 0 μM I194496 group. ^▲^P < 0.05 vs 20 μM I194496 group. (F and H) The effect of I194496 on expression of CSE in MDA-MB-468 cells. *P < 0.05 vs 0 μM I194496 group. ^▲^P < 0.05 vs 24 μM I194496 group. ^Δ^P < 0.05 vs 28 μM I194496 group.

I194496 is a CSE inhibitor obtained by virtual screening based on the crystal structure of CSE protein, as described in our previous paper^20^. To confirm the inhibitory effect of I194496 on CSE, we firstly examined the direct inhibition of CSE by I194496 using the recombinant CSE protein and observed that I194496 possessed significant inhibitory activity against CSE protein with IC_50_ of 0.79 mM (Supplementary Fig. 1C). Then we detected the effect of I194496 on CSE protein concentration in breast cancer cells using the human CSE ELISA kit (Camilo Biological. Inc., Nanjing, China). The results showed that I194496 distinctly reduced the concentrations of CSE protein of MDA-MB-231 and MDA-MB-468 cells in a dose-dependent manner (Fig. 2A,B). Then we further confirmed the inhibitory effect of I194496 on CSE protein activity by the investigation of the production of H_2_S, and we found that I194496 significantly inhibited production of H_2_S in MDA-MB-231 and MDA-MB-468 cells (Fig. 2C,D). In addition, we also found that I194496 significantly decreased the protein and mRNA levels of CSE (Fig. 2E–H and Supplementary Fig. 1) in MDA-MB-231 and MDA-MB-468 cells, but I194496 did not affect the expression of CBS protein (Supplementary Fig. 2E–H) in MDA-MB-231 and MDA-MB-468 cells. The data together indicate that I194496 specifically suppresses the CSE/H_2_S system of TNBC cells. But PAG at the 40 μM concentration had no effect on the production of H_2_S and the concentrations of CSE protein as well as the expression level of CSE protein in TNBC cells (Fig. 2A–D and Supplementary Fig. 2A–D).

### I194496 inhibits the growth, proliferation, migration and invasion of TNBC cells

**Figure 3:**
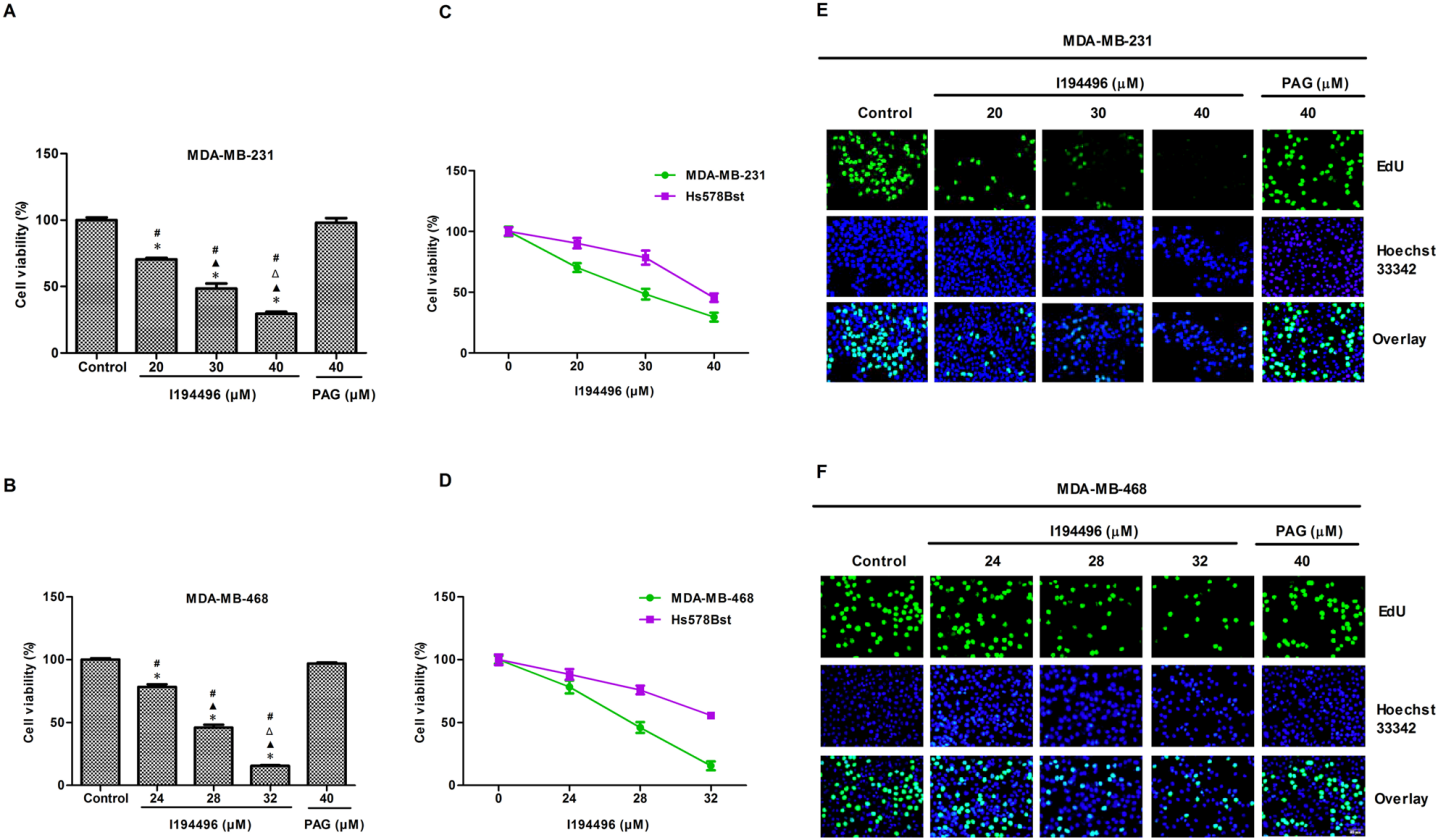
I194496 inhibits the growth and proliferation of TNBC cells. (**A**) MTS assay was used to detect the effect of I194496 on the viability of MDA-MB-231 cells. *P < 0.05 vs Control group. ^▲^P < 0.05 vs 20 μM I194496 group. ^Δ^P < 0.05 vs 30 μM I194496 group. ^#^P < 0.05 vs PAG group. (**B**) MTS assay was used to detect the effect of I194496 on the viability of MDA-MB-468 cells. *P < 0.05 vs Control group. ^▲^P < 0.05 vs 24 μM I194496 group. ^Δ^P < 0.05 vs 28 μM I194496 group. ^#^P < 0.05 vs PAG group. (**C**, **D**) Comparison of the inhibitory effect of I194496 in normal breast epithelial cells and breast cancer cells. (**E**, **F**) EdU assay was performed to detect the proliferation of MDA-MB-231 and MDA-MB-468 cells. The results showed that I194496 decreased the EdU^+^ cell number in a dose-dependent manner. EdU, 5-ethynyl-2′-deoxyuridine. Image magnification, × 200.

**Figure 4:**
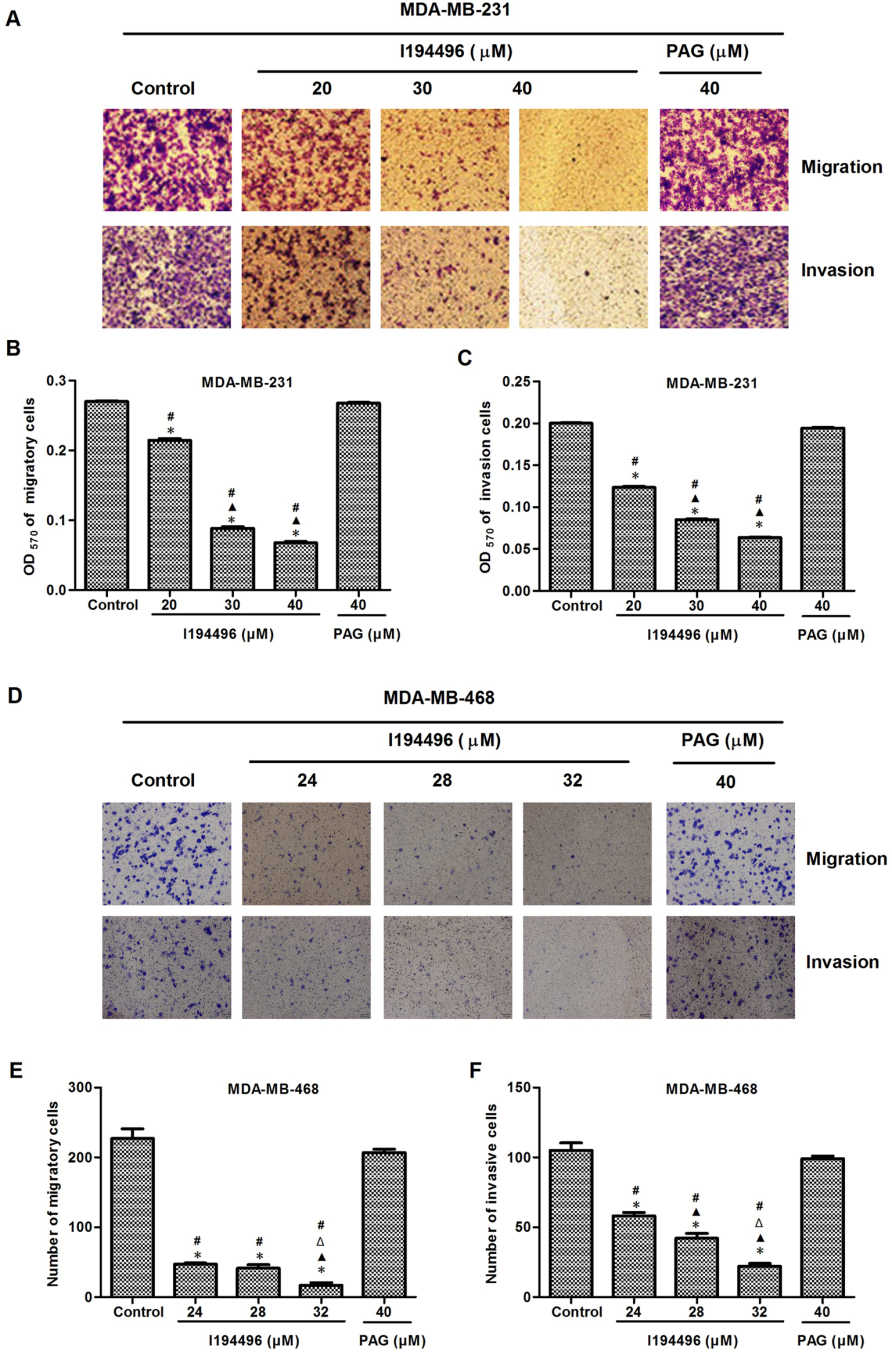
I194496 inhibits the migration and invasion of TNBC cells. (**A**–**C**) The effect of I194496 on the migration and invasion of MDA-MB-231 cells. *P < 0.05 vs Control group. ^▲^P < 0.05 vs 20 μM I194496 group. ^#^P < 0.05 vs PAG group. (**D**–**F**) The effect of I194496 on the migration and invasion of MDA-MB-231 cells. *P < 0.05 vs Control group. ^▲^P < 0.05 vs 24 μM I194496 group. ^Δ^P < 0.05 vs 28 μM I194496 group. ^#^P < 0.05 vs PAG group. Image magnification, × 200.

MTS results showed that I194496 significantly decreased the viability of MDA-MB-231, MDA-MB-468, BT-549 and HCC1937 cells in a dose-dependent manner compared to the control and PAG group (Fig. 3A,B and Supplementary Fig. 3). Moreover, the inhibitory effect of I194496 in normal breast epithelial cells Hs578Bst was lower than that in breast cancer cells (Fig. 3C,D and Supplementary Fig. 3). In the EdU assay, I194496 could markedly decrease the number of EdU^+^ MDA-MB-231, MDA-MB-468, BT-549 and HCC1937 cells in a dose-dependent manner (Fig. 3E,F and Supplementary Fig. 3). In addition, the inhibitory activities of I194496 on cell migration and invasion were also observed in MDA-MB-231 (Fig. 4A–C), MDA-MB-468 (Fig. 4D–F), BT-549 and HCC1937 cells (Supplementary Fig. 4). Taken together, these results indicate that I194496 possesses the capacity of inhibiting the growth, proliferation, migration and invasion of human TNBC cells.

### I194496 blocks the PI3K/Akt and Ras/Raf/ERK signaling pathways in human TNBC cells

**Figure 5:**
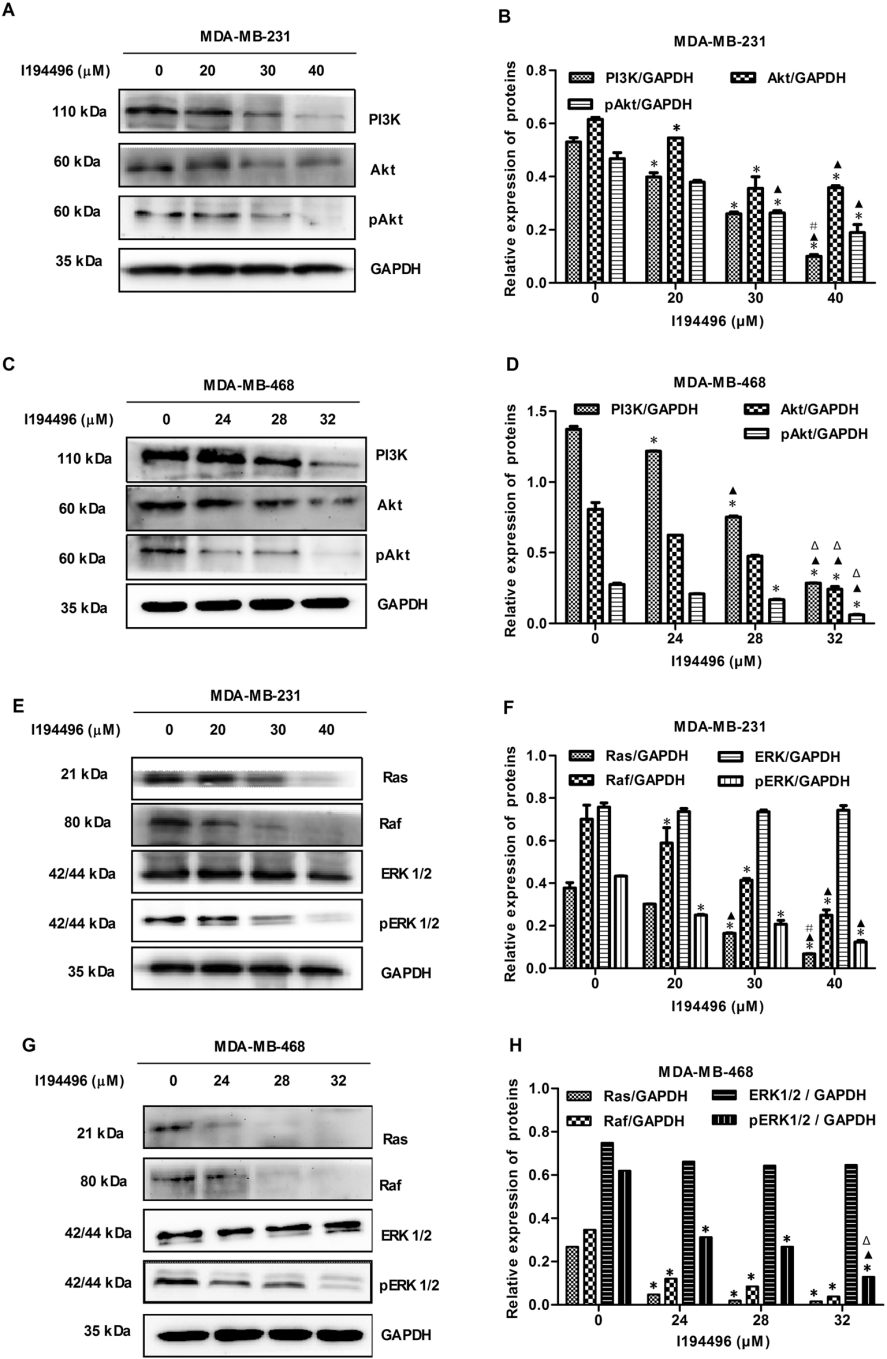
I194496 blocks the PI3K/Akt and Ras/Raf/ERK signaling pathways in human TNBC cells. (**A**, **B**) The effect of I194496 on PI3K/Akt pathway in MDA-MB-231 cells. *P < 0.05 vs 0 μM I194496 group. ^▲^P < 0.05 vs 20 μM I194496 group. ^#^P < 0.05 vs 30 μM I194496 group. (**C**, **D**) The effect of I194496 on PI3K/Akt pathway in MDA-MB-468 cells. *P < 0.05 vs 0 μM I194496 group. ^▲^P < 0.05 vs 24 μM I194496 group. ^Δ^P < 0.05 vs 28 μM I194496 group. (**E**, **F**) The effect of I194496 Ras/Raf/ERK pathway in MDA-MB-231 cells. *P < 0.05 vs 0 μM I194496 group. ^▲^P < 0.05 vs 20 μM I194496 group. ^#^P < 0.05 vs 30 μM I194496 group. (**G**, **H**) The effect of I194496 on Ras/Raf/ERK pathway in MDA-MB-468 cells. *P < 0.05 vs 0 μM I194496 group. ^▲^P < 0.05 vs 24 μM I194496 group. ^Δ^P < 0.05 vs 28 μM I194496 group.

The PI3K/Akt and Ras/Raf/MEK/ERK signaling pathways are commonly dysregulated in breast cancer and activated in TNBC^24,25^. Endogenous or exogenous H_2_S can play their roles by regulating one or both of these pathways^26,27^. In this study, we found that I194496 decreased the levels of PI3K, Akt, and p-Akt proteins in MDA-MB-231 and MDA-MB-468 cells (Fig. 5A–D). Interestingly, we also observed the decreased protein levels of Ras, Raf, and p-ERK in MDA-MB-231 and MDA-MB-468 cells administrated with I194496 (Fig. 5E–H). These data suggest that 194496 may exert anticancer activity by dual targeting of the PI3K/Akt and Ras/Raf/ERK signaling pathway in human TNBC cells.

### I194496 inhibits the Anxa2/STAT3 and VEGF/FAK/Paxillin signaling pathways in human TNBC cells

**Figure 6:**
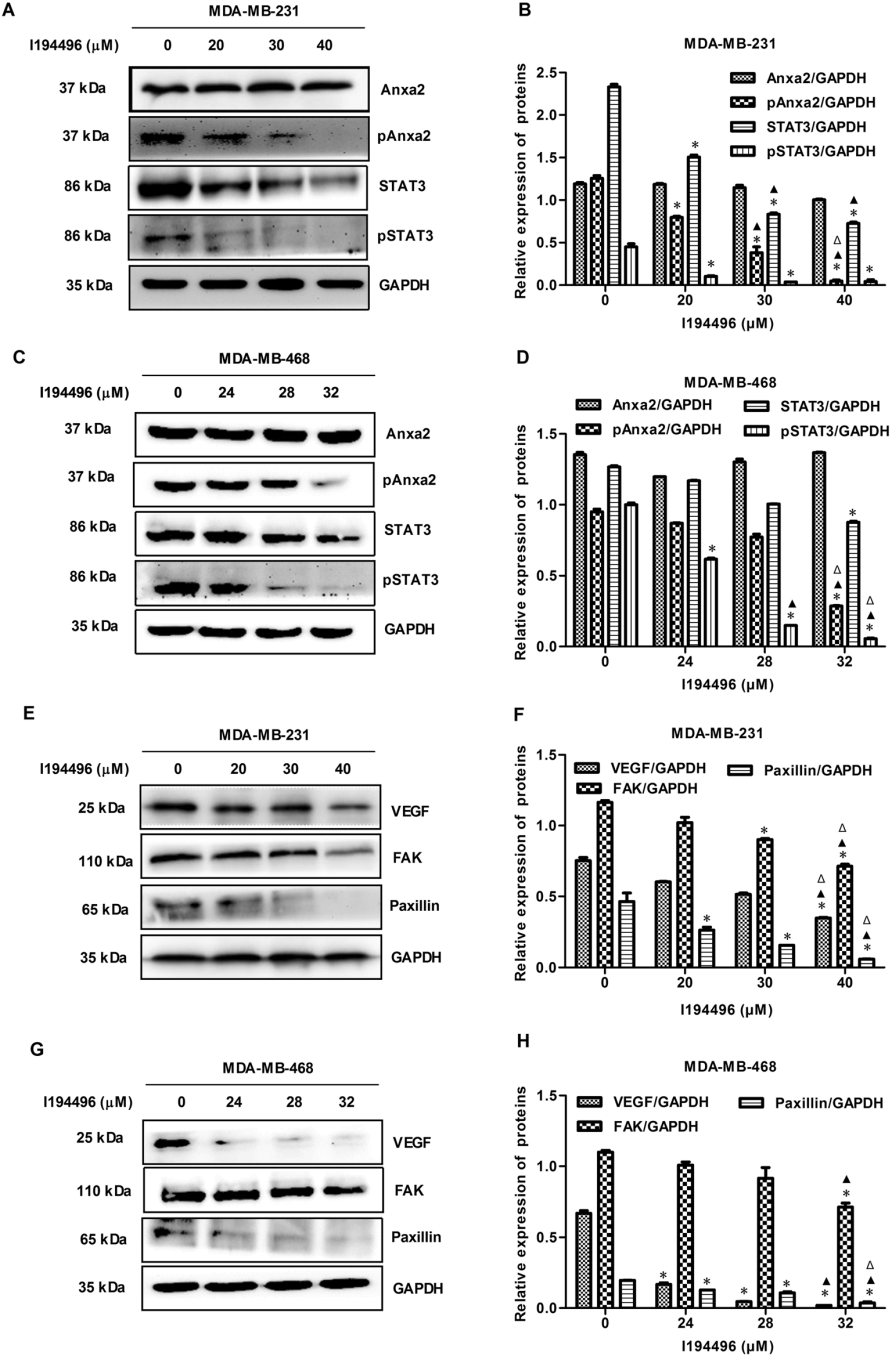
I194496 inhibits the Anxa2/STAT3 and VEGF/FAK/Paxillin signalling pathways in human TNBC cells. (**A**, **B**) The effect of I194496 on Anxa2/STAT3 pathway in MDA-MB-231 cells. ^*^P < 0.05 vs 0 μM I194496 group. ^▲^P < 0.05 vs 20 μM I194496 group. ^Δ^P < 0.05 vs 30 μM I194496 group. (**C**, **D**) The effect of I194496 on Anxa2/STAT3 pathway in MDA-MB-468 cells. ^*^P < 0.05 vs 0 μM I194496 group. ^▲^P < 0.05 vs 24 μM I194496 group. ^Δ^P < 0.05 vs 28 μM I194496 group. (**E**, **F**) The effect of I194496 on VEGF/FAK/Paxillin signaling pathway in MDA-MB-231 cells. ^*^P < 0.05 vs 0 μM I194496 group. ^▲^P < 0.05 vs 20 μM I194496 group. ^Δ^P < 0.05 vs 30 μM I194496 group. (**G**, **H**) The effect of I194496 on VEGF/FAK/Paxillin signaling pathway in MDA-MB-468 cells. ^*^P < 0.05 vs 0 μM I194496 group. ^▲^P < 0.05 vs 24 μM I194496 group. ^Δ^P < 0.05 vs 28 μM I194496 group.

Overexpression of Annexin A2 (Anxa2) is positively correlated with the progression of breast cancer^28^. Moreover, Anxa2 could activate STATS by directly bingding to STAT3, and consequently involve in the invasion and metastasis in breast cancer cells^29^. VEGF is highly expressed in breast cancer cells and VEGF/FAK/Paxillin pathway contributes to the metastasis of breast cancer^19^. Our previous studies have shown that CSE/H_2_S system can promote process and metastasis of breast cancer via the STAT3 signaling pathway and VEGF signaling pathway^18,19^. In this study, we found I194496 decreased the protein levels of p-Anxa2, STAT3, and p-STAT3 in the MDA-MB-231 (Fig. 6A,B) and MDA-MB-468 cells (Fig. 6C,D). Meanwhile, the decreased protein levels of VEGF, FAK, and Paxillin were also observed in MDA-MB-231 and MDA-MB-468 cells administrated with I194496 (Fig. 6E–H). These data implied that I194496 may inhibit metastasis by targeting Anxa2/STAT3 and VEGF/FAK/Paxillin signaling pathways in human TNBC cells.

### I194496 inhibits human TNBC xenograft tumors in nude mice

**Figure 7:**
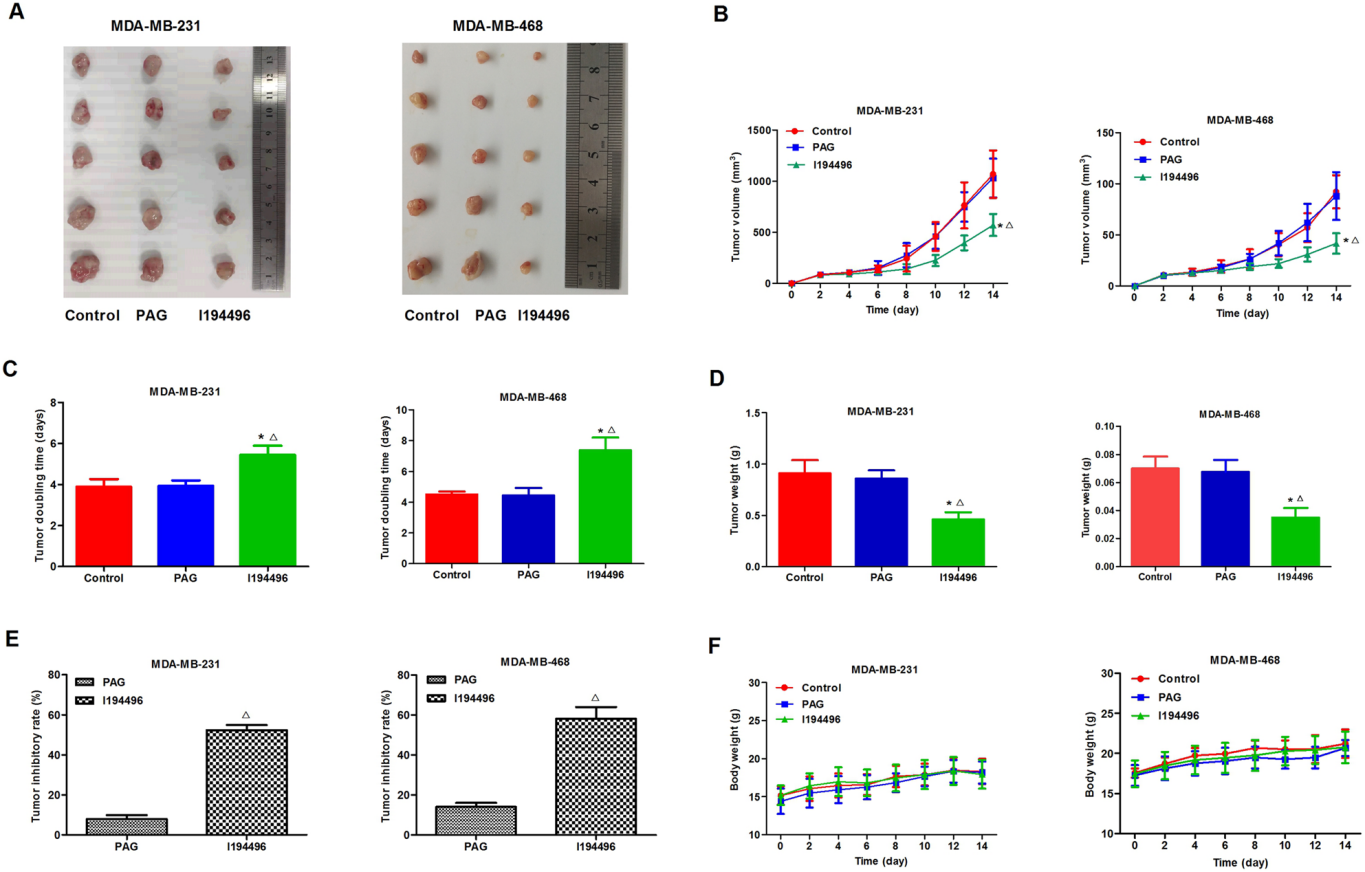
Effects of I194496 on the growth of MDA-MB-231 and MDA-MB-468 xenograft tumors in nude mice. (**A**) The tumors (xenografts) dissected from different groups of nude mice. (**B**, **C**) The tumor volume of each group was measured every other day after tumor growth and the TVDT was calculated by the formula shown in the methods. (**D**, **E**) The tumors dissected from different groups of nude mice were weighed and the inhibition rates of tumor growth were calculated by the formula shown in the methods. (**F**) The body weight change curve of each group during the experiment. All values are presented as mean ± SEM (n = 5); *P < 0.05 compared with the control group; ^△^P < 0.05 compared with PAG group.

**Figure 8:**
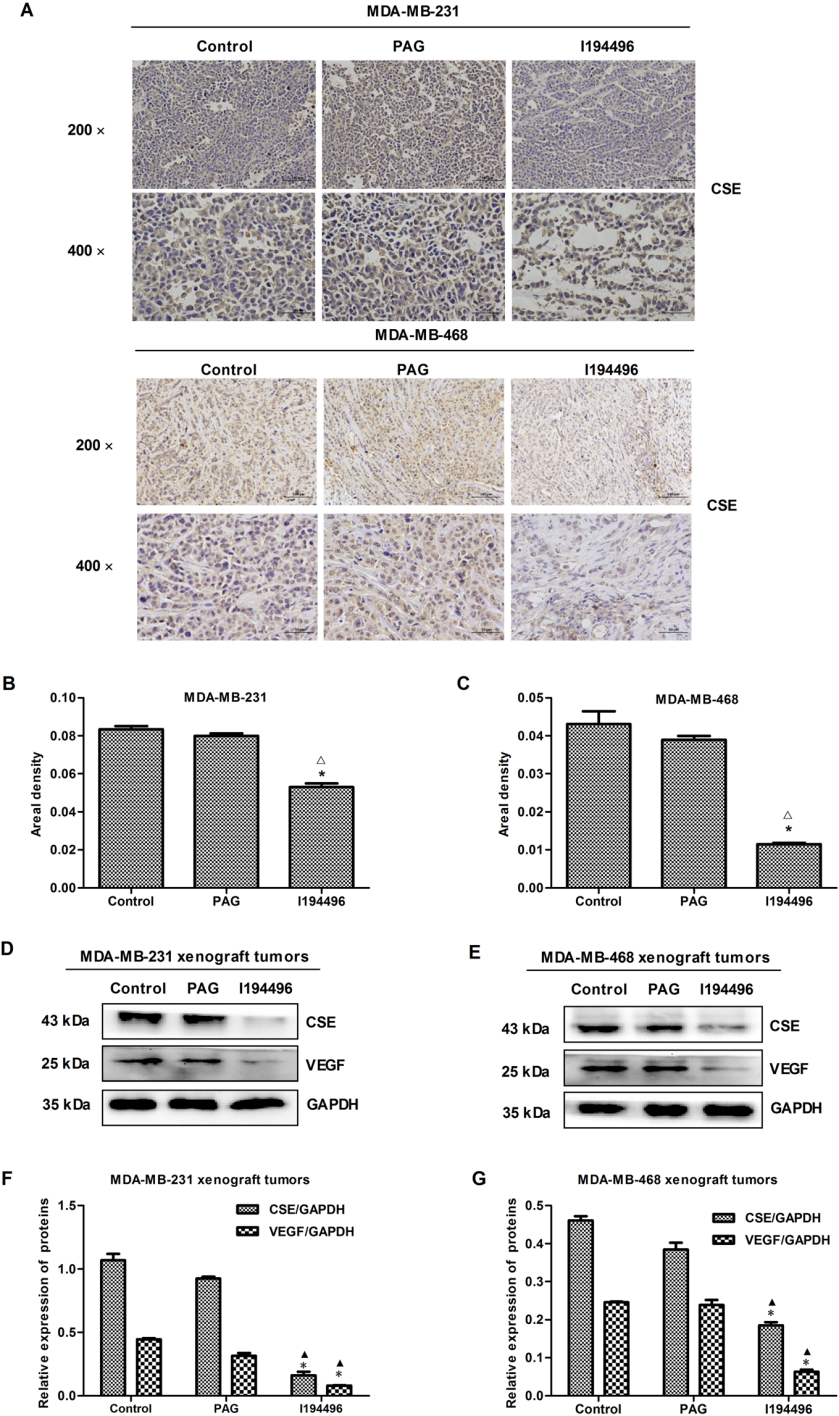
IHC and western blot assays of MDA-MB-231 and MDA-MB-468 xenograft tumors in nude mice. (**A**) Representive photographs of CSE staining in MDA-MB-231 and MDA-MB-468 xenograft tumors; original magnification ×200 and ×400. (**B**, **C**) CSE-positive expression level was calculated as areal density = IOD/AREA, where IOD represents positive cumulative optical density values and AREA represents the pixel area of the organization. Values are presented as mean ± SEM (n = 5); *P < 0.05 compared with the control group; ^△^P < 0.05 compared with PAG group. (**D**, **E**) CSE and VEGF expression analysis in MDA-MB-231 and MDA-MB-468 xenograft tumors. (**F**, **G**) The statistical analysis of CSE and VEGF expression level in MDA-MB-231 and MDA-MB-468 xenograft tumors; Values are presented as mean ± SEM (n = 5); *P < 0.05 compared with the control group; ^▲^P < 0.05 compared with PAG group.

In this part, the effect of I194496 on the growth of TNBC xenograft tumors was investigated. I194496 inhibited the growth of TNBC xenograft tumors compared with the control and PAG group (Fig. 7A–E), but I194496 did not affect the body weight (Fig. 7F). IHC with the CSE antibody confirmed that the in vivo inhibitory effect of I194496 on CSE protein (Fig. 8A–C). Western blot with the CSE antibody further confirmed that the in vivo inhibitory effect of I194496 on CSE protein (Fig. 8D–G). Western blot with the VEGF antibody indicated the possible in vivo inhibitory effect of I194496 on angiogenesis. The data suggest that I194496 may possess the ability of inhibiting the growth and angiogenesis of human TNBC xenograft tumors.

## Discussion

Many works have been performed to obtain new predictive markers and therapeutic targets for the TNBC^30–33^. In our previous study, we found that CSE, an endogenous H_2_S synthase, promoted the progression of breast cancer^18^ and the metastasis of TNBC MDA-MB-231 cells^19^. As a consequence, CSE could be a new therapeutic target in TNBC, and then the study of the inhibitors targeting CSE protein may be of great significance for TNBC.

In this research, we explored the action and mechanism of a new CSE inhibitor I194496 obtaining by virtual screening according to the crystal structure of CSE protein by our research group in the early stage, in TNBC. The data of activity analysis of CSE recombinant protein, CSE ELISA and H_2_S production were integrated to our analysis to provide support to the new compound I194496 as CSE inhibitor. CSE mRNA and protein levels and H_2_S production were shown to be distinctly reduced in TNBC cells administrated with I194496. Subsequently, we researched the actions of I194496 in four TNBC cell lines by MTS, EdU and transwell assays and observed that I194496 significantly decreased the cell viability, the number of EdU^+^ cells as well as the number of migration and invasion in four TNBC cell lines in a dose-dependent manner. It is thus clear that the new CSE inhibitor I194496 could effectively inhibit the growth, proliferation, migration and invasion of TNBC cells. The combined data enhanced our occasions of discerning CSE inhibitor I194496 with real potential to become new therapeutic agent for TNBC.

Furthermore, the possible mechanisms of I194496 against TNBC were explored to give further support to I194496 as a new therapeutic agent. Cell survival and cell proliferation in human breast cancers are associated with the PI3K/Akt signaling pathway or the Ras/Raf/ERK signaling pathway^24,25^. It was found that inhibition of one of the two pathways can still result in the maintenance of the other pathway. So the dual targeting of the two pathways may lead to superior efficacy and better clinical outcome in selected patients. H_2_S exhibits the function of activating the PI3K/Akt and ERK1/2 signaling pathways^34^. Moreover, it is reported that H_2_S regulates the growth of human breast cancer cells or thyroid carcinoma cells through PI3K/Akt/mTOR and RAS/RAF/MEK/ERK signaling pathways^27,35^. In our present study, we observed that both PI3K/Akt signaling pathway and Ras/Raf/ERK signaling pathways were blocked by I194496 in human TNBC cells, which indicated that I194496 could depress growth and proliferation of TNBC cells through dual targeting the PI3K/Akt and Ras/Raf/ERK signaling pathways via inhibiting the release of H_2_S by CSE.

High-expression of Anxa2 could directly regulate STAT3 and consequently enhance the invasion and metastasis in breast cancer cells^28,29^. We here found I194496 decreased the protein levels of p-Anxa2 and p-STAT3, which suggested that the inhibition of Anxa 2/STAT3 pathway may be a way for I194496 to control metastasis of TNBC. VEGF/FAK/Paxillin pathway contributes to the metastasis of breast cancer^19^. Our previous research suggested that CSE expression regulated VEGF expression in MDA-MB-231(19). In this research, VEGF/FAK/Paxillin pathway was inhibited by I194496 in MDA-MB-231 and MDA-MB-468 cells. It follows that the downregulation of VEGF/FAK/Paxillin pathway could be another way for I194496 to inhibit metastasis of TNBC.

Finally, we determined the effects of I194496 on the growth of TNBC xenograft tumors in nude mice. I194496 possessed more strong inhibitory effect on the growth of human TNBC xenograft tumors compared with PAG group. But I194496 did not affect the body weight of mice bearing tumor. Moreover, our data proved that I194496 decreased the level of CSE and VEGF protein in TNBC xenograft tumors. The results indicated that the novel CSE inhibitor could possess more potent anticancer effects through the inhibition of proliferation and angiogenesis in TNBC xenograft tumors.

**Figure 9:**
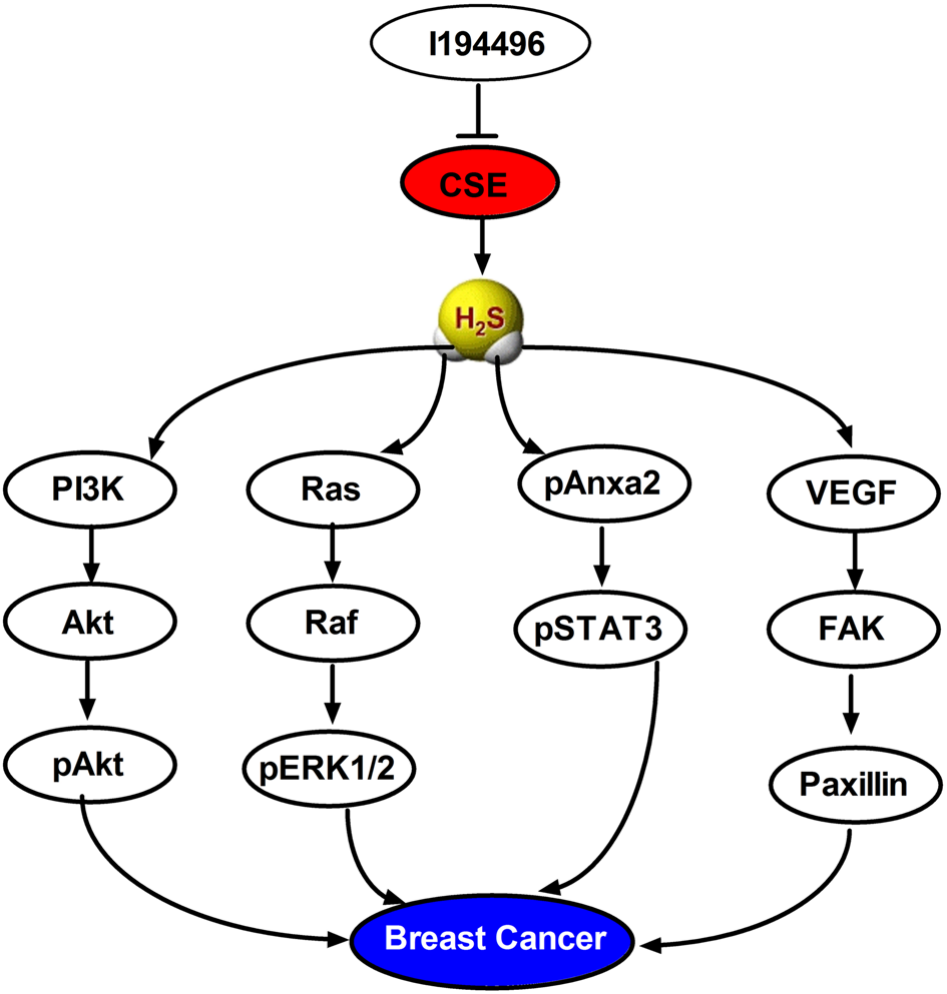
Schematic diagram of the mechanism underlying the inhibitory effects of I194496 on the progression of TNBC. Blunted arrows indicate inhibition.

In conclusion, the novel CSE inhibitor I194496 could restrain the growth of human TNBC cells by dual targeting the PI3K/Akt and Ras/Raf/ERK signaling pathways and also could inhibit the metastasis of human TNBC cells by inhibiting the Anxa2/STAT3 and VEGF/FAK/Paxillin signaling pathways, as shown in Fig. 9. CSE inhibitors might be of novel and potential agents in the treatment of breast cancer.

## Supplementary Information

Supplementary Figures.

## Data Availability

All data and material are available.
