# Supplementary material for: A novel cystathionine γ-lyase inhibitor, I194496, inhibits the growth and metastasis of human TNBC via downregulating multiple signaling pathways
Source: Sci Rep. 2021 Apr 26;11:8963. doi: 10.1038/s41598-021-88355-9 (PMC8076300; doi:10.1038/s41598-021-88355-9)

Title Page

Title:

A novel cystathionine γ-lyase inhibitor, I194496, inhibits the growth and metastasis of human TNBC via downregulating multiple signaling pathways

Ya Liu1 ＃ , Lupeng Wang1 ＃ , Xiuli Zhang2, Yuying Deng1, Limin Pan1, Hui Li3*, Xiaoyan Shi1*, and
Tianxiao Wang1*
1 School of Pharmacy, Henan University, Kaifeng, Henan 475004, P.R. China;
2 Department of Botany, Liaoning Agricultural College, Yingkou, Liaoning 115009, P.R. China;
3 School of Basic Medical Sciences, Joint National Laboratory of Antibody Drug Engineering, Henan University, Kaifeng, Henan 475004, P.R. China.
＃ Equal contributors.
* Corresponding authors.
Hui Li, School of Basic Medical Sciences, Joint National Laboratory of Antibody Drug Engineering, Henan University, Kaifeng, Henan 475004, P.R. China. E-mail: 10190053@vip.henu.edu.cn
Xiaoyan Shi, School of Pharmacy, Henan University, Jinming Road, Kaifeng, Henan
475004,
P.R.
China. E-mail: xiaoyanshi@henu.edu.cn
Tianxiao Wang, School of Pharmacy, Henan University, Jinming Road, Kaifeng, Henan
475004,
P.R.
China. E-mail: **wtx1975@126.com**
Senior author:
Tianxiao Wang
ORCID: 0000-0003-4038-2050Supplementary figure 1

Supplementary figure 1

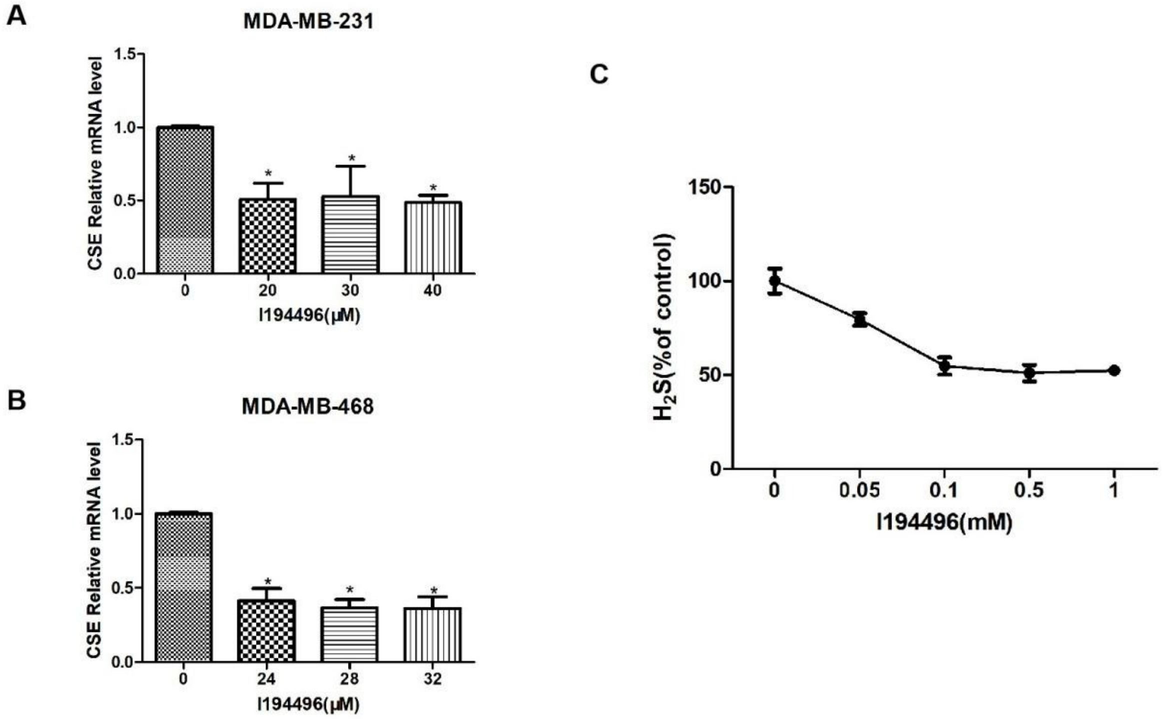

Supplementary figure 1. (A-B) The effect of I194496 on mRNA expression levels of CSE gene in MDA-MB-231 cells and MDA-MB-468 cells.The results showed that I194496 can effect the mRNA of CSE gene but not in a concentration - dependent manner. * $P<0.05$ vs 0 μM I194496 group.(C) The effect of I194496 on the recombinant CSE protein.The results showed that I194496 possessed significant inhibitory activity against CSE protein with IC50 of 0.79mM.

Supplementary figure 2

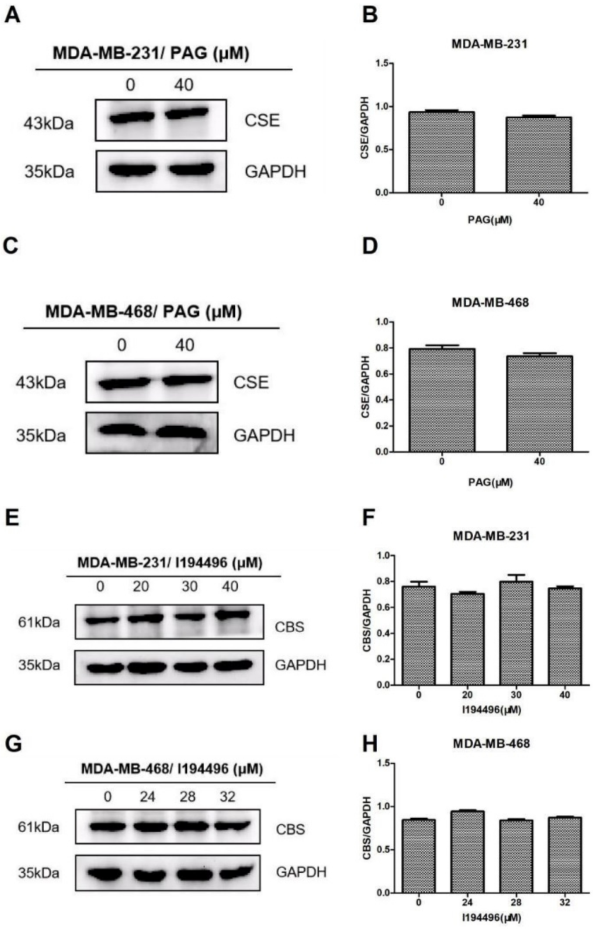

Supplementary figure 2. (A-B) The effect of PAG on CSE expression in MDA-MB-231 cells. (C-D) The effect of PAG on CSE expression in MDA-MB-468 cells. (E-F) The effect of I194496 on CBS expression in MDA-MB-231 cells. (G-H) The effect of I194496 on CBS expression in MDA-MB-468 cells. The results showed that 40 μM PAG did not affect the expression of CSE protein. Meanwhile, the novel CSE inhibitor I194496 did not inhibit the expression of CBS, which suggested that I194496 was specific for CSE.

Supplementary figure 3

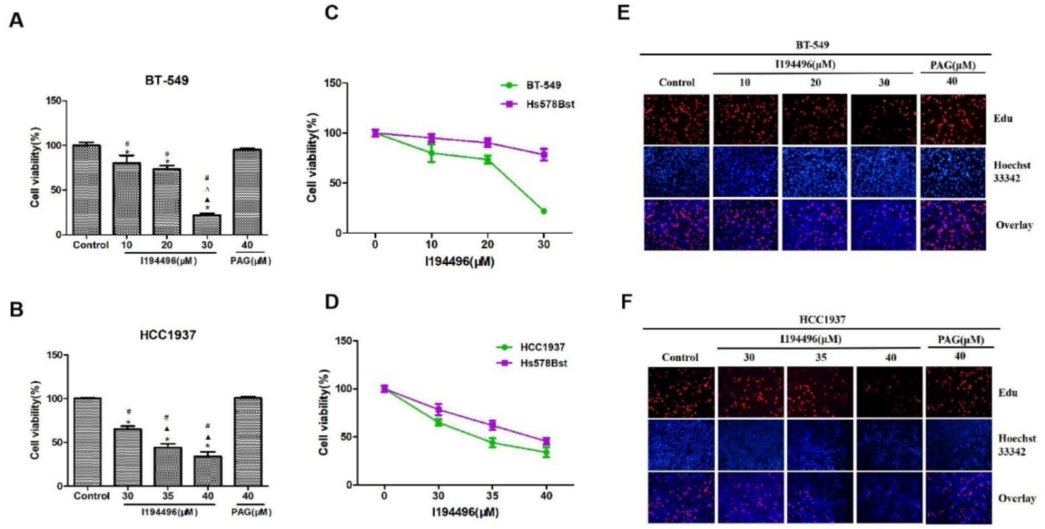

Supplementary figure 3. (A-B) I194496 inhibits the growth and proliferation of TNBC cells. (A) MTS assay was used to detect the effect of I194496 on the viability of BT-549 cells. *P<0.05 vs Control group. ▲P<0.05 vs 10 μM I194496 group. △P<0.05 vs 20 μM I194496 group. #P<0.05 vs PAG group. (B) MTS assay was used to detect the effect of I194496 on the viability of HCC1937 cells. *P<0.05 vs Control group. ▲P<0.05 vs 30 μM I194496 group. #P<0.05 vs PAG group. (C-D) Comparison of the inhibitory effect of I194496 in normal breast epithelial cells and breast cancer cells. (E-F) EdU assay was performed to detect the proliferation of BT-549 and HCC1937 cells. The results showed that I194496 decreased the EdU+ cell number in a dose-dependent manner. EdU, 5-ethynyl-2'-deoxyuridine. Image magnification, x200.

Supplementary figure 4

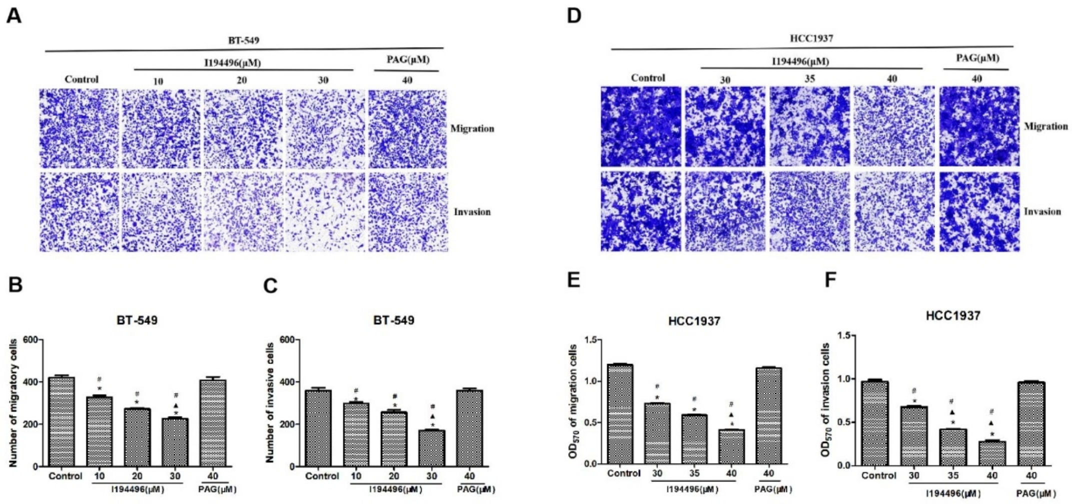

Supplementary figure 4. (A-B) I194496 inhibits the migration and invasion of TNBC cells. (A-C) The effect of I194496 on the migration and invasion of BT-549 cells. *P<0.05 vs Control group. ▲P<0.05 vs 10 μM I194496 group. #P<0.05 vs PAG group. (D-F) The effect of I194496 on the migration and invasion of HCC1937 cells. *P<0.05 vs Control group. ▲P<0.05 vs 30 μM I194496 group. #P<0.05 vs PAG group. Image magnification, x200.

Multiple exposure images

Figure 2 repeat 1

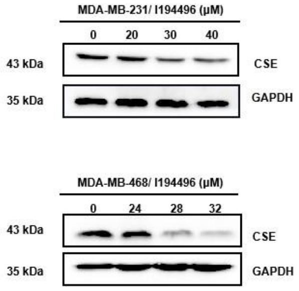

Figure 2 repeat 2

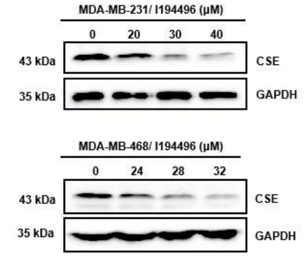

Figure 5 repeat 1

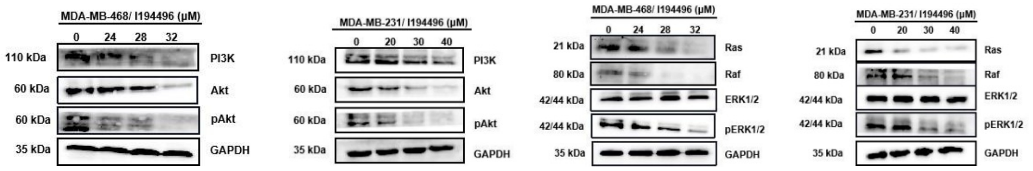

Figure 5 repeat 2

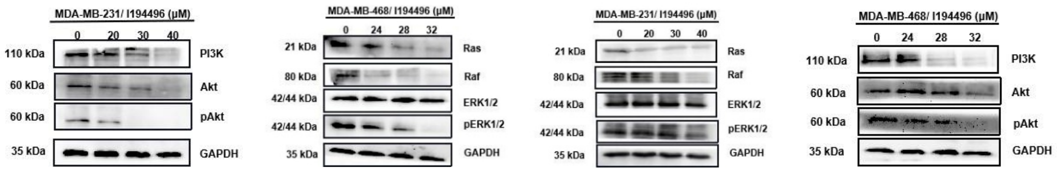

Figure 6 repeat 1

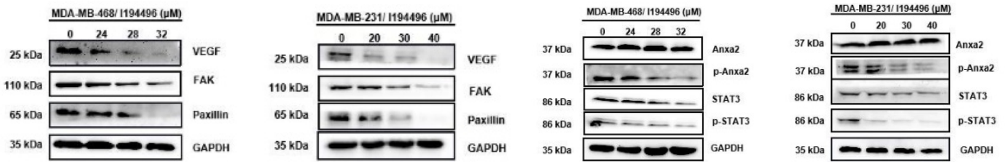

Figure 6 repeat 2

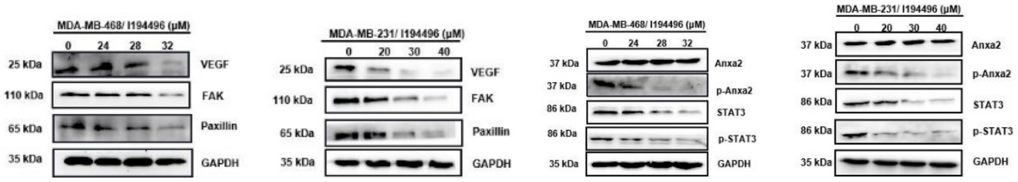

Figure 8 repeat 1

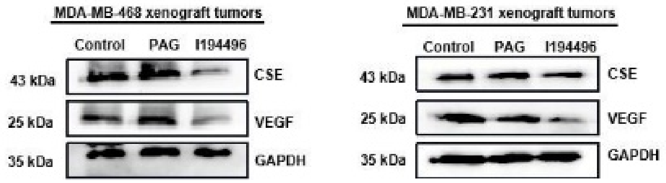

Figure 8 repeat 2

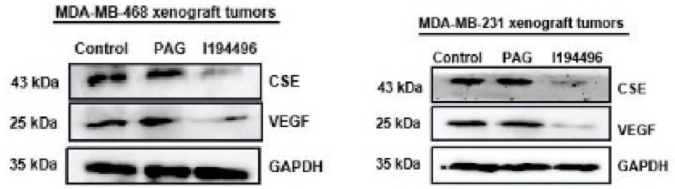

Supplementary figure 2 repeat 1

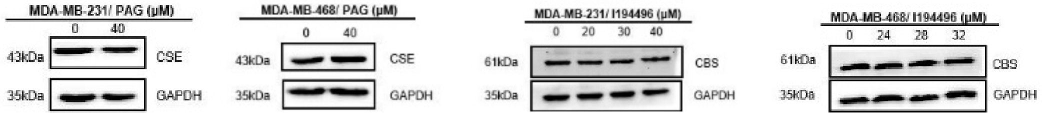

Supplementary figure 2 repeat 2

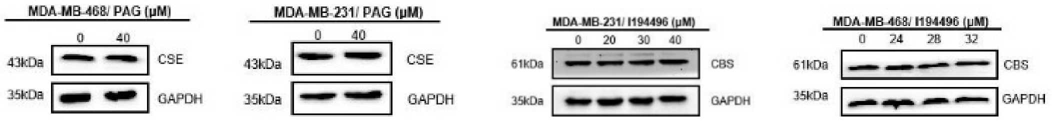

Supplement: Supplementary file 1 — Supplementary Figures. [file 41598_2021_88355_MOESM1_ESM.pdf]
